# What Is the Economic Burden of Subsidized HIV/AIDS Treatment Services on Patients in Nigeria and Is This Burden Catastrophic to Households?

**DOI:** 10.1371/journal.pone.0167117

**Published:** 2016-12-02

**Authors:** Enyi Etiaba, Obinna Onwujekwe, Kwasi Torpey, Benjamin Uzochukwu, Robert Chiegil

**Affiliations:** 1 Department of Health Administration and Management, College of Medicine, University of Nigeria, Enugu Campus, Enugu, Nigeria; 2 Health Policy Research Group, Department of Pharmacology and Pharmaco-Therapeutics, College of Medicine, University of Nigeria, Enugu Campus, Enugu, Nigeria; 3 Strengthening Integrated delivery for HIV/AIDS services (SIDHAS), FHI 360, Plot 1073 J.S. Tarka Street, Garki, P.M.B, Abuja, Nigeria; 4 Department of Community Medicine, College of Medicine, University of Nigeria, Enugu Campus, Enugu, Nigeria; UNAIDS, GUYANA

## Abstract

**Background:**

A gap in knowledge exists regarding the economic burden on households of subsidized anti-retroviral treatment (ART) programs in Nigeria. This is because patients also incur non-ART drug costs, which may constrain the delivery and utilisation of subsidized services.

**Methods:**

An exit survey of adults (18+years) attending health facilities for HIV/AIDS treatment was conducted in three states in Nigeria (Adamawa, Akwa Ibom and Anambra). In the states, ART was fully subsidized but there were different payment modalities for other costs of treatment. Data was collected and analysed for direct and indirect costs of treatment of HIV/AIDS and co-morbidities’ during out-and in-patient visits. The levels of catastrophic health expenditure (CHE) were computed and disaggregated by state, socio-economic status (SES) and urban-rural location of the respondents. Catastrophic Health Expenditure (CHE) in this study measures the number of respondents whose monthly ART-related household expenditure (for in-patient and out-patient visits) as a proportion of monthly non-food expenditure was greater than 40% and 10% respectively.

**Results:**

The average out-patient and in-patient direct costs were $5.49 and $122.10 respectively. Transportation cost was the highest non-medical cost and it was higher than most medical costs. The presence of co-morbidities contributed to household costs. All the costs were catastrophic to households at 10% and 40% thresholds in the three states, to varying degrees. The poorest SES quintile had the highest incidence of CHE for out-patient costs (p<0.0001). Rural dwellers incurred more CHE for all categories of costs compared to urban dwellers, but the costs were statistically significant for only outpatient costs.

**Conclusion:**

ART subsidization is not enough to eliminate economic burden of treatment on HIV patients. Service decentralization to reduce travel costs, and subsidy on other components of HIV treatment services should be introduced to eliminate the persisting inequitable and high cost burden of ART services. Full inclusion of ART services within the benefit package of the National Health Insurance Scheme should be considered.

## Background

HIV/AIDS is a major threat to public health in Nigeria. The annual Acquired Immune Deficiency Syndromes (AIDS) related deaths in Nigeria increased from 192,000 in 2008 to 217,148 in 2012 with a corresponding rise in new infections from 336,379 to 388, 864 in the same period. [1] A World Health Organization (WHO) update shows that currently, 7.9 million people are receiving treatment for HIV/AIDs in SSA (Sub-Saharan Africa) and 1.5 million of these reside in Nigeria.[2] The overall prevalence of HIV/AIDS is 4.1% in Nigeria, although rates vary across the six geopolitical zones in the country with the highest prevalence in the North Central Zone (7.5%) and the lowest prevalence rate in the North Western Zone, at 2.1%.[1]

Although drugs for HIV/AIDS are free for patients at designated health facilities and ART centres in Nigeria, evidence suggests that expenditures on HIV/AIDS care and treatment can be catastrophic to patients and their households due to costs of other components of care. Patients pay for non- ART drugs for opportunistic infections, non- routine tests, medical consultations, transportation, feeding and hospital stays. [3,4] The costs of these often have impoverishing effects on some households and hinder them from getting the necessary care[3] [4], especially in settings like Nigeria where payment for healthcare remains predominantly through out-of-pocket (OOP) payments.[5]

The problem of provision of free ART is currently confounded by dwindling government income in Nigeria due to the falling oil price, leading to reductions in government health budgets that may translate to the possibility of introducing user fees for HIV/AIDs treatment.[6] The implication of introducing fees for service on the economic burden of HIV/AIDS is not known and there is poor evidence of the economic burden of even free services on different population groups in Nigeria.

This paper adds new knowledge to what is known, on the current level of residual economic burden, and the catastrophic health expenditures on households of free ART treatment services amongst different population groups. It is hoped that the evidence will assist policy makers and programme managers in both government and non-governmental organizations (NGOs) in the design of strategies to reduce or remove financial burdens to HIV/AIDS patients for all population groups.

## Methods

### Study Sites

The study was undertaken in Adamawa, Akwa Ibom and Anambra states in the north-east, south-south and south-east geopolitical zones of Nigeria, respectively. These three states were chosen to obtain sub-nationally representative data as well as highlight the differences in economic burden as a result of HIV/AIDS treatment in the various geographic areas.

Prevalence of HIV in the states vary: Adamawa (3.8%), Akwa Ibom (10.9%) and Anambra (8.7%)[1]; and the 2006 census estimated their various population as follows: Adamawa (3,178,950), Akwa Ibom (3,902,051) and Anambra (4,177.828).[7]

Six (6) purposively selected Local Government Areas (LGAs) from the three states were included in the study; 1 urban LGA (state capital) and 1 rural LGA that was easily accessible from the state capital and have ART treatment centres with a high patient load, as follows: Adamawa state (Yola North and Numan), Akwa Ibom state (Uyo and Etinan), Anambra (Awka South and Anaocha). In Adamawa state 2 facilities (1 rural and 1 urban) were sampled, in Akwa Ibom State, 3 facilities (1 rural, 2 urban), and in Anambra state, 3 facilities (2 rural, I urban) were sampled.

While ART was fully subsidized in the three study states, there were within and between state differences in costs of other treatment components. Across the states, patients paid for their laboratory investigations and any incidental co-morbidity costs. In Adamawa, some facilities also fully subsidized treatment of opportunistic infections (OIs) while others charged a fee. In Akwa Ibom state, all patients were routinely charged a fee for treatment of OIs, while treatment for OIs was fully subsidized in Anambra state. Co-morbidities are considered as other illnesses which are not necessarily due to an individual being HIV positive. The common illnesses considered in this study were malaria and other febrile illnesses. OIs are those conditions that are likely due to diminished immunity as a result of the individual suffering from HIV/AIDS.

### Study design and data collection

Pre-tested interviewer-administered questionnaires elicited information on demographic details of respondents, details of treatment seeking, direct costs of treatment (registration, consultation, investigations, treatment, transport, food, accommodation and caregiver costs) and indirect costs of treatment (productivity loss of patients and caregivers). The costs were those incurred on out-patient and in-patient visits for HIV/AIDS. In addition, data was collected on household expenditures on food and non-food items to enable the computation of catastrophic costs. Hence, data was collected on household monthly expenditures on clothing, house rent, household furniture (if bought within one year of survey), cooking fuel, healthcare, educational expenses and other expenses such as electricity bills.

Sample size per LGA was estimated at 10% precision to determine a 50% effect at 95% confidence interval. This gave an initial estimated sample size of 96, which was rounded up to 100 and increased to 250 to enable a valid sub-group analysis, a total of 1500 interviews across the states.

Target respondents were adult patients (18 years and above) with HIV/AIDS who attended and sought treatment from the selected facilities and had consented to participate in the survey. Anonymized informed consent was obtained from willing participants, following which information was collected by trained interviewers. Participants were consecutively recruited until the estimated sample size was reached. Interviews were conducted with participants after they had been seen by a health worker on the day of interview. If they were given a prescription for drugs or requests for any form of investigation, the participants would have to collect the drugs and undergo the requested investigations so that all costs incurred will be accurately collected and documented. The challenges to this were that sometimes, participants spent a long time during these processes and were tired and sometimes impatient during the interviews. To limit this, participants were asked a second time if they were still willing to be interviewed after they had gone through the consultation, investigations and collection of drugs; and only those still willing were then interviewed.

### Data analysis

The variables analysed were demographic characteristics, treatment seeking, direct and indirect costs (direct:-direct medical, direct non-medical and indirect costs:-productivity losses) and catastrophic health expenditure (CHE). Due to the variations in treatment modalities between states, data was disaggregated by states. We also analysed the differences in treatment and cost variables amongst SES groups and between urban and rural dwellers for further inquiry into the level of inequity in the system. An asset-based SES index was created. Information was also collected on estimated monthly income, monthly non-food expenditure including all other health expenditure. Monthly income was not included in the generation of SES index because, contextually this has been found not be very reliable due to the fact that it is common to have multiple sources of income and secondly, household heads are not always willing to declare their income. Principal Component Analysis was used to generate the SES quintiles.

Direct costs were computed as direct medical costs (registration, consultation, investigations-x-rays and laboratory tests, drugs) and direct non-medical costs (transportation, feeding, accommodation, caregiver and other costs). Indirect costs (productivity losses) were computed as the number of days fully or partially missed from work by study participants or other household member. These were converted to money using the national minimum wage of N18, 000.00 ($111.11) per month as standard. In costing, for conversion of the local currency (Naira) to US dollars, the exchange rate of the dollar to Naira at the time of the study was $1 (USD) = N162.0.[8] All costs are expressed first in Nigerian Naira and followed by their equivalent in United States Dollars (USD) in brackets.

An indirect cost was computed based on the national minimum wage of $111.11 per month and assuming 20 effective working days per month.

The incidence of CHE was estimated by measuring the number of respondents whose monthly ART-related household expenditure (for in-patient and out-patient visits) as a share of monthly non-food expenditure was greater than 40% and 10% respectively. The monthly non-food expenditure by households was computed as a total of what they spent on clothing, house rent, household furniture (if bought within one year of survey), cooking fuel, healthcare, educational expenses, other expense e.g. electricity bills. The two thresholds were chosen for the results to be comparable to existing literature. A previous study reported the existence of catastrophic expenditure for almost all patients on out-patient visits and for all costs associated with ART.[9]

The frequency distributions of categorical variables were calculated and means calculated for continuous variables. The Kruskal-Wallis non-parametric test, which reports a Chi-square (Chi^2^) statistic, was used to compare differences in means of the cost variables. Equity ratios were also computed in examining SES differences in the key variables.

### Ethical Considerations

Ethical approval was obtained from the Institutional Review Board of Family Health International (FHI) 360, the Protection of Human Subject Committee, North Carolina, U.S.A and the Health Research Ethics Committee of the University of Nigeria Teaching Hospital, Ituku-Ozalla, Enugu state, Nigeria. Participants provided informed consent and were reassured of total anonymity. Consent was provided by participants ticking a designated consent box after having read the information sheet and asked for any clarifications. This method of consent was agreed to by both ethical boards named above. Participants were also assured that their participation in the study or lack of it will not affect the services and treatment received for their illness and that the only disadvantage to them will be the time spent during the interview and this will be kept to the minimum possible duration. Names and addresses were not collected from participants.

## Results

### Respondent characteristics

We enrolled a total of 1, 557 patients in Adamawa, Akwa Ibom and Anambra states between 13^th^ September 2013 to 10^th^ November 2013.

Table 1 shows details of the socio-demographic characteristics of respondents. There were more females (68.3%) and also more female heads of households in total, with variation across the three states. Over 50% of respondents were monogamously married. The mean age of respondents across the states was 37.1 years and the average number of residents in each household was 5.1. Over 90% of respondents had obtained one form of education or the other. The mean number of years spent schooling was 10.8 years. Adamawa respondents were multi-ethnic in contrast with Anambra and Akwa Ibom states where the predominant ethnic groups were Igbo (95.0%) and Ibibio (86.4%) respectively. Respondents were almost equally divided across the SES quintiles in the three study states.

**Table 1:** Key socio-demographic characteristics of survey respondents, by state.

Variables	Categories of variables	Adamawa n (%)	Akwa Ibom n (%)	Anambra n (%)	Total N (%)	Missing data
Gender (Total)	Total	**507 (100.0)**	**514 (100.0)**	**536 (100.0)**	**1,557 (100.0)**	0
	Male	150(29.6)	155(30.2)	188(35.1)	493(31.7)	
	Female	357(70.4)	359(69.8)	348(65.0)	1,064(68.3)	
Status in household	Total	**507 (100.0)**	**514 (100.0)**	**536 (100.0)**	**1,557 (100.0)**	0
	Male Head	140(27.6)	139(27.0)	171(31.9)	450(28.9)	
	Female Head	113(22.3)	136(26.1)	271(50.7)	520(33.4)	
	Son/Daughter	84(16.6)	115(22.4)	71(13.3)	270(17.3)	
	Other	170(33.5)	123(23.9)	23(4.3)	316(20.3)	
**Marital status**	Total	**506 (100.0)**	**514 (100.0)**	**535 (100.0)**	**1,555 (100.0)**	2
	Monogamous	238(47.0)	226(44.0)	348(65.1)	812(52.2)	
	Polygamous	41(8.1)	5(1.0)	8(1.5)	54(3.5)	
	Single	87(17.2)	146(28.4)	80(15.0)	313(20.1)	
	Divorced	25(4.9)	18(3.5)	5(0.9)	48(3.1)	
	Separated	27(5.2)	28(5.5)	19(3.6)	73(4.7)	
	Widowed	89(17.6)	91(17.7)	75(14.0)	255(16.4)	
**Mean Age in yrs (sd)**		36.7(9.8)	35.2(10.1)	39.3(9.7)	37.1(10.0)	
**Attended school**		411(81.2)	490(95.3)	512(95.5)	1,413(90.8)	
**Average Number of years spent schooling**		10.7 (5.8)	10.5(3.8)	11.2(4.3)	10.8 (4.5)	
**Ethnic group**	Total	**504 (100.0)**	**514 (100.0)**	**534 (100.0)**	**1,552 (100.0)**	5
	Hausa	24(4.8)	6(1.2)	13(2.4)	43(2.8)	
	Igbo	10(2.0)	10(2.0)	507(95.0)	527(34.0)	
	Yoruba	4(0.8)	1(0.2)	1(0.2)	6(0.4)	
	Fulani	35(6.9)	0(0.0)	1(0.2)	36(2.3)	
	Marghi	8(1.6)	0(0.0)	0(0.0)	8(0.5)	
	Ibibio	2(0.4)	444(86.4)	0(0.0)	448(28.9)	
	Efik	0(0.0)	4(0.8)	3(0.6)	7(0.5)	
	Other	421(83.5)	49(9.5)	7(1.3)	477(30.7)	
**Employment status**	Total	**507 (100.0)**	**514 (100.0)**	**535 (100.0)**	**1,556 (100.0)**	1
	Government	78(15.4)	43(8.9)	45(8.1)	168(10.8)	
	Private sector	24(4.7)	32(6.7)	58(104)	113(7.3)	
	Self employed	331(65.3)	338(69.8)	382(68.7)	1,056(67.9)	
	Retired	7(1.4)	9(1.9)	3(0.5)	19(1.2)	
	Unemployed	56(11.1)	44(9.1)	57(10.3)	159(10.2)	
	Student	6(1.2)	12(2.5)	7(1.3)	25(1.6)	
	Other	4(0.8)	6(1.2)	4(0.7)	15(1.0)	
**Residence**	Total	**506 (100.0)**	**514 (100.0)**	**535 (100.0)**	**1,555 (100.0)**	2
	Urban	249 (49.2)	286 (55.6)	259 (48.4)	794 (51.1)	
	Rural	257 (50.8)	228 (44.4)	276 (51.6)	761 (48.9)	
**SES quintiles**	Total	**506(100.0)**	**514(100.0)**	**536(100.0)**	**1556 (100.0)**	1
	Quintile 1 (Most poor)	163(32.2)	69(13.4)	80(14.9)	312(20.05)	
	Quintile 2 (Poor)	104(20.6)	104(20.2)	103(19.2)	311(19.99)	
	Quintile 3 (Average)	95(18.8)	110(21.4)	106(19.8)	311(19.99)	
	Quintile 4 (Least poor)	71(14.0)	109(21.2)	131(24.4)	311(19.99)	
	Quintile 5 (Rich)	73(14.4)	122(23.7)	116(21.6)	311(19.99)	

### Treatment characteristics: Out-Patient services

Most respondents had been receiving HIV/AIDS treatment; a minority (5.2%) attending the facility for the first time on the day of interview. Across the study states, check- up appointments were predominantly three monthly. More than half of respondents (56.2%) in Akwa Ibom state and over two thirds in Adamawa and Anambra states had been on treatment for more than one year. Most respondents utilized public transport to attend the facilities for their appointments. (Table 2)

**Table 2:** Health seeking pattern for out patient and inpatient visits across the states.

OUT-PATIENT VISIT							IN-PATIENT VISIT						
Variable	Category	Adamawa N = 507 n (%)	Akwa Ibom N = 514 n (%)	Anambra N = 536 n(%)	Chi^2^ (P value)	Total N = 1557	Variable	Category	Adamawa N = 506 n(%)	Akwa Ibom N = 513 n(%)	Anambra N = 536 n(%)	Chi2(p-value)*	Total 1,555^**^
**First visit**	Yes	19 (3.8)	40(7.8)	26 (4.8)	**8.6 (0.013)**	81(5.2)	**Admitted in past 3 months**	Yes	33 (6.5)	20 (3.9)	16 (3.0)	**8.2 (0.02**)	69 (4.4)
	No	488 (96.2)	474 (92.2)	510 (95.2)		1481 (94.2)	**No. of adm. in past 3mths** Mean (S.D.)		1.5 (0.9)	1.1 (0.3)	1.1 (0.3)		1.3 (0.7)
**How long on ART?**	Total	**492 (100.0)**	**479 (100.0)**	**507 (100.0)**	**55.3 (0.000)**	**1,478 (100.0)**	**Facility admitted**	Private	11 (34.4)	5 (27.8)	2 (14.3)	**15.52 (0.004)**	18 (28.1)
	<6months	44 (8.9)	90 (18.8)	77 (15.2)		211 (14.3)		Public	20 (60.6)	5 (27.8)	8 (57.1)		33 (51.6)
	>6months	32 (6.5)	63 (13.1)	25 (4.9)		120 (8.1)		Mission	1 (3.0)	8 (44.4)	4 (28.6)		12 (18.8)
	Up to1yr	63 (12.8)	59 (12.3)	46 (9.1)		168 (11.3)		PMV	1 (3.0)	0 (0.0)	4 (28.6)		1 (1.6)
	>1yr	353 (71.8)	267 (55.7)	359 (70.8)		979 (66.2)							
**Freq. of visit**	Weekly	13 (2.6)	8 (1.7)	7 (1.4)	**844 (0.000)**	28 (1.9)							
	Monthly	78 (15.8)	168 (35.2)	63 (12.4)		309 (20.9)							
	Every 2m	111 (22.6)	246(51.6)	174 (34.1)		531 (35.9)							
	Every 3m	267 (54.3)	18 (3.8)	36 (7.1)		321 (21.7)							
	Every 6m	3 (0.6)	1(0.2)	0 (0.0)		4 (0.3)							
	Yearly	0 (0.0)	1(0.2)	0 (0.0)		1 (0.1							
	No answer	0 (0.0)	35 (7.3)	226 (44.3)		261 (17.7)							
	Other	19 (3.9)	0 (0.0)	4 (0.8)		23 (1.6)							
**Mode of transport**	Total	**505 (100.0)**	**514 (100.0)**	**536 (100.0)**	**37.31(0.000)**	**1,555 (100.0)**	**Mode of transport**					**3.56 (0.8)**	
	Motorcycle	132 (26.1)	194 (37.7)	138 (25.8)		464 (29.8)		Motorcycle	10 (31.3)	7 (31.3)	3 (18.8)		18 (27.7)
	Public transport	341 (67.5)	293 (57.0)	373 (69.6)		1,007 (64.8)		Public transport	17 (53.1)	8 (56.3)	8 (62.5)		33 (50.8)
	Private transport	14 (2.7)	23 (4.5)	17 (3.2)		54 (3.5)		Private transport	4 (9.4)	3 (12.5)	3 (18.8)		13 (20.0)
	Walk	17 (3.3)	4 (0.8)	7 (1.3)		28 (1.8)		Walk	2 (6.3)	0 (0.0)	0 (0.0)		1 (1.5)
	Other	1 (0.2)	0 (0.0)	1 (0.2)		2 (0.1)		Other	0 (0.0)	0 (0.0)	0 (0.0)		0 (0.0)
**Treatment received**							**Treatment received**						
	Routine drugs	506 (99.8)	491 (95.5)	510 (95.3)	**21.52 (0.000)**	1,507 (96.9)		Routine drugs	10 (30.3	11 (61.1)	2 (14.3)	**8.31 (0.02)**	23 (35.4)
	Drugs for TB	1 (0.2)	20 (3.9)	16 (2.9)	**16.32 (0.000)**	37 (2.4)		Drugs for TB	1 (3.2)	4 (22.2)	1 (7.1)	**5.05(0.22)**	6 (9.4)
	Other OI’s	118 (23.3)	192 (37.4)	356 (66.4)	**208.36 (0.000)**	666 (42.8)		Other OI’s	21 (63.6)	9 (50.0)	3 (25.0)	**7.0 (0.03)**	33 (51.6)
	Lab test	3 (0.6)	34 (6.6)	41 (7.7)	**31.43 (0.000)**	78 (5.0)		Lab test	7 (21.2)	13 (72.2)	2 (14.3)	**16.58 (0.000**)	22 (33.9)
	Co-morbidities	9 (1.8)	24 (4.7)	23 (4.3)	**7.27 (0.03)**	56 (3.6)		Co-morbidities	10 (34.5)	11 (38.0)	8 (27.6)	**6.81 (0.03)**	29 (100.0)

PMV-patent medicine vendor

*fischer’s exact

** missing data = 2

At their hospital visits, almost all respondents (96.9%) received routine drugs for HIV/AIDS as appropriate, few received treatment for tuberculosis (2.4%) and almost half (42.8%) were treated for OIs across the states. However, treatment for OIs was a lot higher than average in Anambra (66.4%) and much lower in Adamawa (23.3%) state. A few respondents also had laboratory tests carried out (5.0%); this was higher in Anambra state (7.7%) and lowest in Adamawa state (0.6%). About 3.6% of respondents were treated for co-morbidities, the most common being malaria treatment.(Table 2)

### Treatment characteristics: In-Patient services

Sixty nine (4.4%) respondents reported having been admitted at least once to one facility or the other within the three months prior to the study but predominantly in a public facility (P = 0.01). Most patients spent less than 3 weeks but a few spent over a month (P = 0.08).The predominant reason for admissions was for treatment of opportunistic infections across the three study states (P = 0.03). In addition, 29 respondents (46.8%) were also treated for co-morbidities. Adamawa state had the highest number of admissions in the three months preceding the study (P = 0.02), but there was a higher proportion of co-morbidities and laboratory testing in respondents admitted in Akwa Ibom state and these were statistically significant (P = 0.03, P = 0.000).

### Direct Costs of Treatment

#### Out-patient visit

Table 3 shows a distribution of mean out-patient costs among all respondents irrespective of whether they incurred costs or not. Respondents incurred a mean direct medical cost of N141.37 ($0.87), a mean direct non-medical cost of N740.43 ($4.57) and a mean total out-patient cost of N881.8 ($5.44) across the three states. At an average of four visits per year, this gives an annual total cost estimate of N3,527.2 ($21.76) [$5.44 per visit multiplied by four visits]. Direct medical and direct non- medical costs were highest in Akwa Ibom state (p<0.0001). Besides registration cost which was highest in Anambra state (p = 0.0001), all component costs were highest in Akwa Ibom state. The most expensive component of the direct non-medical cost was transportation cost.

**Table 3:** Mean treatment costs per out-patient visit for All respondents–Naira [USD].

Variable	Category	Adamawa N = 507	Akwa-Ibom N = 514	Anambra N = 536	Combined N = 1557	Chi Square (Pr)*
**Direct medical costs**	Total	**30.10 [0.19]**	**254.86 [1.57]**	**137.79 [0.85]**	**141.37 [0.87]**	**37.72[0.0001]**
	Registration	15.27 [0.09]	64.40 [0.40]	97.94 [0.60]	59.95 [0.37]	20.28[0.0001]
	Consultation	0.32 [0.001]	1.36 [0.008]	0 [0.0]	0.55 [0.003]	0.037[0.98]
	Tests	11.66 [0.07]	21.50 [0.13]	26.60 [0.16]	20.05 [0.12]	0.153[0.93]
	Drugs	2.86 [0.018]	167.61 [1.03]	13.25 [0.08]	60.82 [0.38]	33.27[0.0001]
**Direct non-medical costs**	Total	**723.94 [4.47]**	**891.69 [5.50]**	**610.98 [3.77]**	**740.43 [4.57]**	**38.82 [0.0001]**
	Transport	618.00 [3.81]	775.64 [4.79]	520.49 [3.21]	636.30 [3.93]	42.87[0.0001]
	Food	99.58 [0.61]	109.63 [0.68]	88.04 [0.54]	98.92 [0.61]	8.68[0.01]
	Accommodation	2.21 [0.01]	0.58 [0.004]	0.56 [0.003]	1.10 [0.007]	0.04[0.98]
	Caregiver	4.17 [0.03]	5.84 [0.04]	1.89 [0.01]	3.94 [0.02]	0.10[0.94]
**Total outpatient cost**		**754.04 [4.65]**	**1146.55 [7.08]**	**748.77 [4.62]**	**881.8 [5.44]**	**100.14 [0.0001]**

*kruskal wallis

When costs are distributed only among respondents who actually incurred costs for one item or the other, it was found that the mean direct medical cost across the three states increased to N395.28 ($2.44) while the mean direct non-medical costs increased to N748.44 ($4.62).

#### In-patient stays

Table 4 shows that the means costs; direct medical costs, direct non-medical costs and total in patient costs were highest in Akwa Ibom state. For individual components, patients in Adamawa state paid more for registration_N841.38 ($5.19) while patients in Akwa Ibom state paid more for consultation and drugs [N1800.0($11.11), N12,250.0 ($75.62)] respectively. Cost of drugs was the most expensive component of the direct medical costs. Patients in Akwa Ibom state also incurred the most costs among the direct non- medical components, though only food, transportation and accommodation costs were statistically significant. The total mean cost for an in-patient stay was highest in Akwa Ibom state at N41,915.56 ($258.74) and lowest in Adamawa state at N10,133.03 ($62.55) (p = 0.0001).

**Table 4:** Mean treatment cost for an in-patient visit–Naira [USD]. (N = 69).

Variable	Category	Adamawa = 33	Akwa-Ibom = 20	Anambra = 16	Combined = 69	Chi sq.(P)**
**Direct medical costs (N = 56)** *	Total	**6390.63 [39.45]**	**16855.56 [104.05]**	**7566.67 [46.71]**	**9880.36 [60.99]**	**14.56[0.0007]**
	Registration (N = 52) *	841.38 [5.19]	717.65 [4.43]	241.67 [1.49]	731.73 [4.52]	24.07[0.0001]
	Consultation (N = 21) *	340.63 [2.10]	1800.0 [11.11]	0 [0]	688.10 [4.25]	8.35[0.0032]
	Test (N = 29) *	3435.0 [21.20]	4407.14 [27.20]	3950.0 [24.38]	3922.07 [24.21]	4.31[0.116]
	Drugs(N = 48) *	4867.69 [30.05]	12250 [75.62]	10000 [61.73]	8063.75 [49.78]	10.82 [0.004]
**Direct non-medical costs**	Total	**3963.06 [24.46]**	**25060 [154.69]**	**7237.0 [44.67]**	**10710.49 [66.11]**	**26.94[0.0001]**
	Transport (N = 59) *	703.87 [4.34]	826.67 [5.10]	742.0 [4.58]	747.80 [4.62]	6.74[0.03]
	Food (N = 49) *	2397.04 [14.80]	7259.38 [44.81]	2908.33 [17.95]	4047.36 [24.98]	16.77[0.0002]
	Accommodation	1323.08 [8.17]	20003.33 [123.48]	14666.67 [90.54]	11653.23 [71.93]	17.08[0.0002]
	Caregiver: (N = 27) *	1452.78 [8.97]	2857.14 [17.64]	1750 [10.80]	1838.89 [11.35]	3.80[0.15]
**Total in-patient cost (N = 61)** *		**10133.03 [62.55]**	**41915.56 [258.74]**	**11777.0 [72.70]**	**19780.98 [122.10]**	**24.58[0.0001]**

*available cost data

**kruskal wallis

### Differences in out-patient and in-patient costs by state, SES and urban/rural residence

Table 5 shows that both out-patient and in-patient costs were highest in Akwa Ibom state (p = 0.0001). The most-poor quintile (Q1) incurred the highest out-patient costs while the poor quintile (Q2) incurred the highest in-patient costs but none of these were statistically significant. Urban residents incurred more out-patient costs (p<0.0001), as well as in-patient costs, but in-patient costs was not statistically significant.

**Table 5:** Categorization of Mean costs for an Out-patient and In-patient expenditures- Naira [USD].

Variable	Category	Out-patient N = 1557	In- Patients N = 69
State	Adamawa: N = 507	763.07 [4.71]	10133.03 [62.55]
	Akwa-Ibom:N = 514	1153.28 [7.12]	41915.56 [258.74]
	Anambra: *N = 536*	754.4 [4.66]	11777.0 [72.70]
	[P-value]*	**100.12 [0.0001]**	**24.58 [0.0001]**
**SES**	Q1: N = 312	984.77 [5.86]	5899.17 [36.41]
	Q2: N = 310	862.97 [5.33]	20836.36 [128.62]
	Q3: N = 311	907.21 [5.57]	19518.0 [120.48]
	Q4: N = 311	820.0 [5.06]	46912.22 [289.58]
	Q5:N = 311	870.61 [5.37]	11360 [70.12]
	[P-value]*	**5.78 [0.22]**	**7.16 [0.13]**
Residence	Urban: N = 794	915.02 [5.65]	26022.86 [160.63]
	Rural: N = 761	863.5 [5.33]	14484.85 [89.41]
	[P-value]*	**18.55 [0.0001]**	**1.06 [0.3]**

*Kruskal-Wallis

### Catastrophic Health Expenditure (CHE)

Table 6 shows that at 40% threshold, Adamawa state had the highest incidence of CHE for both in-patient and out-patient expenditures. At 10% threshold Adamawa also has the highest out-patient CHE while Akwa Ibom state had the highest incidence for in-patient CHE (p<0.000). At both thresholds, the poorest quintile (Q1) incurred the highest CHE for out-patient costs (p<0.000). Rural dwellers also incurred more catastrophic payments for all categories of expenditure but these were only significant for outpatient costs. Rural dwellers had the higher incidence of CHE for all costs at both thresholds but only the out-patient costs were statistically significant. (p = 0.000)

**Table 6:** Catastrophic health expenditures on HIV/AIDS treatment at two thresholds.

Variable	Category	OP expenditure >40% of non-food expenditure n (%)	IP expenditure >40% of non- food expenditure n (%)	OP expenditure >10% of non- food expenditure n (%)	IP expenditure >10% of non-food expenditure n (%)
**State**	A**da**mawa	50 (9.9)	27(81.8)	216 (42.6)	32(96.8)
	Akwa Ibom	39 (7.6)	17(94.1)	230 (44.8)	18(100.0)
	Anambra	33 (6.2)	5(60.0)	198 (36.9)	7 (90.0)
	**Total**	**122(7.8**)	**49(3.2)**	**646 (41.5**)	**57(96.7)**
	Chi Sq.(Pr)*	**5.01 (0.08)**	**16.55(0.000)**	**7.19(0.03)**	**18.55(0.000)**
**SES quintile**	Q1	54 (17.3)	10(91.7)	192 (61.5)	11 (100.0)
	Q2	30(9.7)	11(100.0)	148 (47.6)	11 (100.0)
	Q3	9(2.9)	16(84.2)	119(38.3)	19(100.0)
	Q4	17(5.5)	7(75.0)	105 (33.9)	9(100.0)
	Q5	12 (3.9)	5(50.0)	79(25.4)	7(80.0)
	**Total**	**122 (7.9)**	**49(81.7**)	**645(41.5)**	**57(96.7)**
	Chi sq. (Pr)*	**59.89(0.000)**	**7.4(0.11)**	**98.4(0.000)**	**7.56(0.11)**
**Residence**	Urban	41 (5.2)	21(77.8)	236(29.7)	24(92.6)
	Rural	80 (10.5)	28(84.9)	407 (53.6)	33(100.0)
	**Total**	**122(7.9)**	**49(81.7)**	**643(41.4)**	**57(96.7)**
	Chi sq.(Pr)*	**15.55 (0.000**)	**1.37(0.24)**	**90.9(0.000)**	**1.91(0.17)**

*Pearson’s

### Cost of co-morbidities

The cost of out-patient co-morbidities was highest in Adamawa state, amongst females, urban residents, and amongst the least poor (Q4). For in-patient services, respondents in Anambra state incurred the highest co-morbidity costs. However, none of these costs was statistically significant.

### Indirect Costs of Treatment

About a third of respondents (30.4%) reported having missed at least one entire day of work in the previous three months due to illness with a mean of 9.7 (14.0) days. It was found that 30% of respondents reported an average of 8 days of limited work output by them or by a household member due to respondents’ illness. The number of days of ‘limited work’ for respondents or household member was highest in Akwa Ibom state but this was not statistically significant. More rural respondents missed entire days of work (p = 0.003), more household members of rural respondents also missed entire days of work (p<0.0001). The loss in wages for an entire missed day of work was N900.72 ($5.56) per day. Half of this amount was used to estimate limited work days. The highest cost per respondent for missed days of work was incurred in Adamawa state-N10,530.0 ($65) and the lowest in Akwa Ibom state-N7,920.18 ($48.89).

## Discussion

The findings show that although the cost of antiretroviral drugs are essentially free, it is not enough to provide financial risk protection as patients still bear some costs which include payments for other medical conditions such as opportunistic infections and co-morbidities; and other direct non-medical costs such as transportation cost. This was evident in Akwa Ibom state where patients incurred the highest direct medical costs as a result of having to pay for treatment of all opportunistic infections. This high cost burden of hospitalization has also been found in India where there has been a high rate of hospitalization for opportunistic infections.[10]

Direct non-medical costs still impose a substantial economic burden on individuals and their households and the major cost drivers were transportation and food. Transport cost alone was more than three times the total medical costs, suggesting that patients still have to travel far to get to where ART services are delivered. This can potentially hinder effective service delivery as patients may ignore or postpone check-up appointments when cost of transportation to the treatment facility poses a barrier. In Ghana, total out- patient expenditure on ART was found to be up to US$ 55 depending on how far the patients had to travel to get to the nearest ART centre and how long they had to wait at the ART facility.[11]

Despite subsidization of ART services, it remains inequitable for the rural dwellers and their household members who incurred substantial amounts of cost burden as a result of missing entire days of work or limited productivity due to illness.

Households still incur CHE at various thresholds since out-of-pocket (OOP) payments still predominate in the health system. In the out-patient departments, where majority of people living with HIV/AIDS (PLWHA) seek treatment, the level of CHE increased as the SES decreased, similar to a previous study in another state[9], with the poorest households suffering the highest catastrophic expenditure for out-patient treatment. The implication of this is that poor households may be driven further into impoverishment[12] as a result of these residual cost burden with an overall negative impact on the achievement of universal health coverage. A similar socio-economic inequality has also been found in South Africa, Spain and some high income countries.[13–16]

The existence of co-morbidities also contributed to the economic burden to PLWHA, even when ART is fully subsidized. Where these co-morbidities result in hospitalization, it leads to lost wages for patients and some household members. In India, a study documented that rates of hospitalization for HIV-infected patients were 8 times higher than those of the uninfected population, and most of these admissions were due to opportunistic infections in patients with advanced disease.[10]

A study limitation was the difficulty posed by collecting reliable estimates of income, hence the national minimum wage was used across board to generate daily equivalent of earning, which may have over-estimated or under-represented the indirect costs incurred by the respondents. This made it difficult to compare the estimated annual expenditure for ART services with patients’ and households’ annual income. Other potential limitations include; the use of a cross-sectional survey design data with a recall period of three months for inpatient stay. In collecting data on household consumption of other goods and services using a one-month recall period, the accuracy could have been limited because expenditures on several items are incurred at different frequencies (daily, monthly, quarterly, and yearly) and may not be captured accurately in a one month period even if the expenditures are annualised. However, this appears to be the most feasible method. A convenience sample of patients making clinic visits is likely to under-estimate the overall burden on households, as those most burdened by costs will make fewer visits. This will be taken into consideration for further studies. Another limitation is that we did not collect data on the number and characteristics of patients who refused to participate in the study and this may have systematically biased the results. However, there were very few refusals.

Further research questions include; the financial impact of HIV/AIDS related costs incurred by households over time and households’ response to these costs; are patients ignoring or postponing symptoms, complying with follow up appointments as a result of these residual costs?

In conclusion, despite free ART services and all the concerted efforts made towards improving treatment of HIV/AIDS, households incur substantial costs in accessing and utilizing ART treatment services. Non-medical expenses such as transport and feeding are substantial and predispose patients to CHE. In-patient visits predispose patients to a high level of CHE and strategies to protect people from in-patient medical expenditures should be explored. In a fragile health system like Nigeria, it appears that subsidizing ART is not enough, since there are other potential barriers to access.

Decentralization of services to the remote areas and integration of services into primary health care will reduce transportation costs. Further subsidy on HIV related laboratory investigations and harmonization of service delivery charges across states to remove the present disparity are also recommended. Financial risk protection especially for the most poor and rural dwellers is important and should be explored if the health system is to be strengthened towards achieving universal health coverage (UHC).[12] Some successes have been recorded in other low and middle income countries like Ghana and Rwanda where health insurance has protected the poor and decreased CHE.[11]
